# Late Breaking Abstracts

**DOI:** 10.1111/ene.70677

**Published:** 2026-06-26

## Monday, June 29 2026

## Late‐breaking Oral Session

## LB_01

### Central disorders of hypersomnolence: linking CSF hypocretin‐1 to symptom severity

#### J. Zhou ^1^, Z. Zhang^2^, J. Gool^1^, R. Fronczek^1^, G. Mayer^3^, M. Partinen^4^, S. Overeem^5^, J. Santamaria^6^, K. Šonka^7^, C. Bassetti^8^, J. Meer^8^, R. Rio‐Villegas^9^, C. Veauthier^10^, S. Miano^11^, R. Peraita‐Adrad^12^, U. Kallweit^13^, E. Feketeova^14^, J. Bušková^15^, C. Donjacour^16^, R. Khatami^2^, G. Lammers^1^

##### ^1^Stichting Epilepsie Instellingen Nederlands (SEIN), Sleep‐wake Center, Heemstede, Netherlands, ^2^Center for Sleep Medicine, Sleep Research and Epileptology, Klinik Barmelweid AG, Barmelweid, Switzerland, ^3^Neurology Department, Hephata Klinik, Schwalmstadt, Germany, ^4^Department of Clinical Neurosciences, Clinicum, University of Helsinki, and Helsinki Sleep Clinic, Terveystalo Healthcare, Helsinki, Finland, ^5^Sleep Medicine Center Kempenhaeghe, Heeze, the Netherlands, ^6^Neurology Service, Institut de Neurociències Hospital Clínic, University of Barcelona, Barcelona, Spain, ^7^Neurology Department and Centre of Clinical Neurosciences, First Faculty of Medicine, Charles University and General University Hospital, Prague, Czech Republic, ^8^Department of Neurology, Inselspital, Bern University Hospital, and University of Bern, Bern, Switzerland, ^9^Neurophysiology and Sleep Disorders Unit, Hospital Universitario Vithas Madrid Arturo Soria, Universidad CEU San Pablo, Madrid, Spain, ^10^Charité – Medical University Berlin, Interdisciplinary Center for Sleep Medicine, Berlin, Germany, ^11^Neurocenter of Southern Switzerland, Faculty of Biomedical Sciences, Università della Svizzera Italiana, Sleep Medicine Unit, EOC, Lugano, Switzerland, ^12^Sleep and Epilepsy Unit – Clinical Neurophysiology Service, University General Hospital Gregorio Marañón and Research Institute Gregorio Marañón, University Complutense of Madrid (UCD), Madrid, Spain, ^13^Center for Narcolepsy and Hypersomnias, Professorship for Narcolepsy and Hypersomnolence Research, Department of Medicine, University Witten/Herdecke, Witten, Germany, ^14^Neurology Department, Medical Faculty of P. J. Safarik University, University Hospital of L. Pasteur Kosice, Kosice, Slovak Republic, ^15^Department of Sleep Medicine, National Institute of Mental Health, Klecany and 3rd Faculty of Medicine, Charles University, Prague, Czech Republic, ^16^Sleep Wake Centre, Stichting Epilepsie Instellingen (SEIN), Zwolle, The Netherlands

**Background and aims:** To identify non‐cataplexy symptoms indicating hypocretin‐1 deficiency, and to investigate the association between CDH symptom presentation and varying CSF hypocretin‐1 levels.

**Methods:** We included 302 CDH individuals from the European Narcolepsy Network database. Hypocretin‐1 levels were categorized as ≤ 40, 41–110, 111–200, and > 200 pg/mL. Machine learning (stochastic gradient boosting) was used to explore non‐cataplexy predictors of hypocretin‐1 deficiency. Logistic and quantile regression models examined associations between absolute hypocretin‐1 levels and demographic, subjective symptom, polysomnography, and multiple sleep latency test (MSLT) measures, adjusted for age, sex, and body mass index. Cataplexy features were analyzed among individuals with cataplexy and hypocretin‐1 levels ≤ 200 pg/mL.

**Results:** Non‐cataplexy variables predicted degrees of hypocretin‐1 deficiency with moderate certainty. In all individuals, lower hypocretin‐1 levels were associated with shorter sleep latency and rapid eye movement sleep latency on both polysomnography and MSLT, more MSLT sleep‐onset rapid eye movement periods, higher Epworth Sleepiness Scale scores, increased periodic limb movement index, and more frequent disturbed nocturnal sleep. Among individuals with cataplexy and hypocretin‐1 levels ≤ 200 pg/mL, lower levels were associated with earlier excessive daytime sleepiness onset, younger age at diagnosis, greater diagnostic certainty of hypnagogic hallucinations and sleep paralysis, and more frequent and complete cataplexy attacks.

**Conclusion:** Lower hypocretin‐1 levels were associated with more severe symptoms, both in the overall CDH population and in individuals with cataplexy and low hypocretin. Even within the deficient range, hypocretin‐1 variation contributed to individual differences in symptom presence and severity.

**Disclosure:** Jingru Zhou reports no competing interests. Zhongxing Zhang received consulting fees from Takeda. Jari Gool did advisory work for and received research support from Takeda and Jazz; received a speaker fee from Pharmanovia; and is a member of a Safety Review Committee for Centessa. Rolf Fronczek did advisory work for and received research support from Takeda and Jazz; received a speaker fee from Pharmanovia; and is a member of a Safety Review Committee for Centessa. Geert Mayer received consulting fees from Takeda; received honoraria for a lecture from Löwenstein Medical; and received support for attending meetings and/or travel from Bioprojet. Markku Partinen reports no competing interests. Sebastiaan Overeem received consulting fees paid to his institution from Bioproject, Takeda, Jazz Pharmaceuticals, and UCB Pharma. Joan Santamaria reports no competing interests. Karel Šonka participated in Alkermes‐sponsored clinical trials. Claudio L.Bassetti received research support from the Swiss National Science Foundation and the University of Bern (IRC Grant); received consulting fees from Takeda and Idorsia; and received honoraria for educational events from Boehringer Ingelheim and Takeda. Julia van der Meer reports no competing interests. Rafael del Río‐Villegas received consultancy fees from Alkermes, Bioprojet, and Takeda; and travel funds from Bioprojet, Jazz, and Takeda. He is a member of the International Board for Alkermes and Takeda. Christian Veauthier serves as Chair of the Berufsverband Deutscher Schlafmediziner (BDS e.V.) and as Secretary of the Deutsche Gesellschaft für Schlafforschung und Schlafmedizin (DGSM e.V.). Silvia Miano reports no competing interests. Rosa Peraita‐Adrados reports no competing interests. Ulf Kallweit received consulting fees from Takeda and Bioprojet; received honoraria for educational lectures from Bioprojet and Pharmanovia; served on advisory boards for Takeda and Bioprojet; and serves as a Board Member of the European Academy of Neurology (EAN) and Vice‐Speaker of the Committee on Hypersomnias of the German Sleep Society (DGSM), both unpaid roles. Eva Feketeova reports no competing interests. Jitka Bušková reports no competing interests. Claire E.H.M. Donjacour reports no competing interests. Ramin Khatami received consulting fees from Takeda and Idorsia; received honoraria for lectures from Idorsia and Takeda; and served on advisory boards or data safety monitoring boards for Takeda and Idorsia. Gert Jan Lammers received consulting fees from Takeda, Bioproject, Alkermes, Jazz, and Daiichi Sankyo; received honoraria for a lecture from Bioproject; and received support for attending meetings and/or travel from Takeda and Bioproject.

## LB_02

### Apathy marks brain network and genetic vulnerability to cognitive decline in Parkinson's disease

#### B. Attaallah

##### Imperial College London, UK

**Background and aims:** Motivational deficits such as apathy and impulse‐control behaviours (ICBs) are common in Parkinson's disease (PD), with increasing evidence suggesting their relevance to cognitive function. However, the mechanisms by which they influence cognitive trajectories remain under investigation.

**Methods:** Using the Parkinson's Progression Markers Initiative (PPMI), we examined apathy and ICBs in relation to longitudinal cognition, resting‐state functional connectivity, structural imaging, and genetic susceptibility in early PD.

**Results:** In 1502 participants at baseline, apathy‐containing phenotypes were comparatively stable over time, whereas isolated ICB was more transient, consistent with a stronger medication‐linked component. Across up to 15 years of follow‐up, higher baseline apathy robustly predicted steeper decline in both latent global cognition and Montreal Cognitive Assessment performance, independent of demographic, motor, depressive, and treatment covariates; ICB‐related effects on decline were weak and not robust. In resting‐state functional MRI (*n* = 310), the Network‐Based Statistic identified an apathy‐related somatomotor–dorsal‐attention connectivity signature independent of disease severity and dopaminergic medication, whereas any ICB‐related connectivity attenuated after levodopa‐equivalent dose adjustment. Lower connectivity within this subnetwork predicted steeper cognitive decline, mediated the apathy–decline association in an age‐dependent manner, and tracked smaller hippocampal volume and faster subsequent atrophy. Apathy‐related cognitive decline was selectively amplified in GBA‐associated PD, with apathetic GBA carriers declining approximately five‐fold faster than non‐apathetic sporadic cases.

**Figure:**
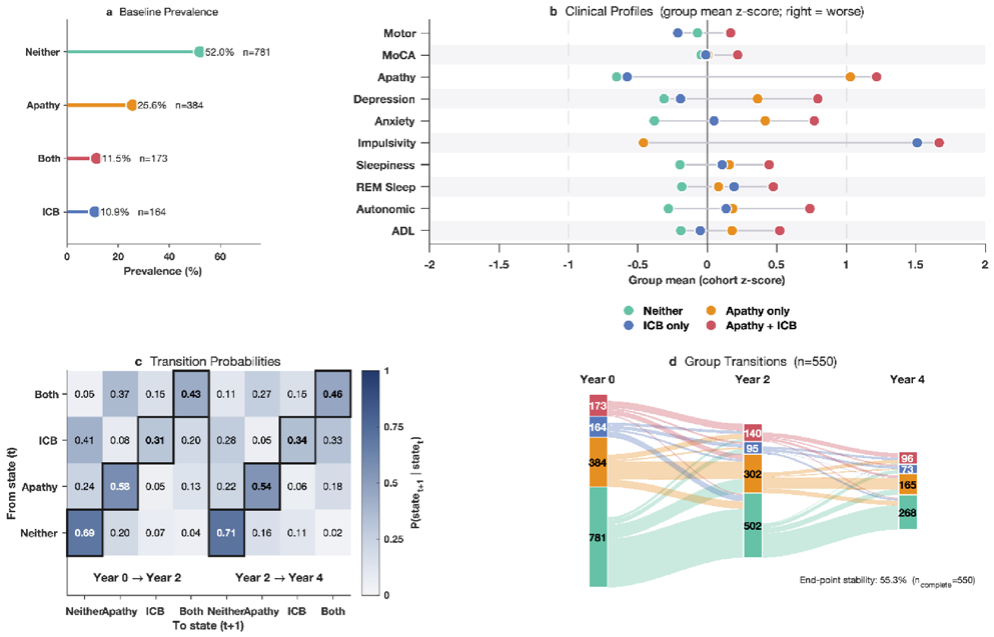
**FIGURE 1** Apathy–ICB co‐occurrence and longitudinal stability in early Parkinson's disease.

**Figure:**
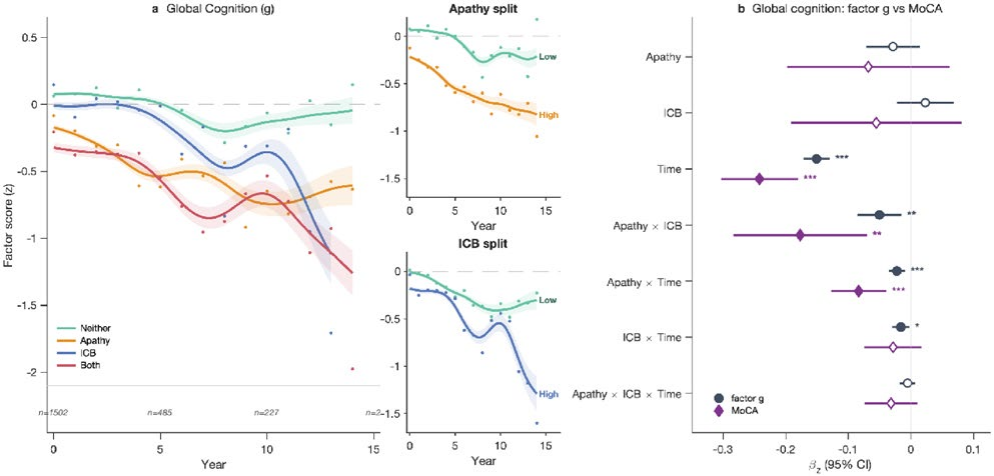
**FIGURE 2** Apathy preferentially predicts faster global cognitive decline in early Parkinson's disease.

**Conclusion:** pathy, rather than ICB severity, identifies a stable and clinically meaningful motivational marker of adverse cognitive trajectory in early PD, linked to a specific connectivity phenotype and amplified by GBA status.

**Disclosure:** None.

## LB_03

### Large multicentre cohort of rare hypomyelinating leukodystrophies supports clinico‐radiological and molecular reappraisal

#### S. Patel, R. Maroofian, H. Houlden

##### Department of Neuromuscular Diseases, National Hospital of Neurology and Neurosurgery, Queen Square, London, UK

**Background and aims:** Hypomyelinating leukodystrophies (HLDs) are traditionally defined radiologically by persistent hypomyelination on brain MRI. However, many rare HLD‐associated genes remain represented only by isolated reports or very small case series, limiting understanding of their full clinical, radiological, and biological spectrum.

**Methods:** We performed retrospective multicentre deep phenotyping and neuroradiological review of individuals with pathogenic variants in several rare HLD‐associated genes, including FAM177A1, AIMP1, AIMP2, HSPD1, EPRS1, and NKX6.2.

**Results:** Cross‐cohort analysis identified reproducible clinical subgroups, including classic Pelizaeus–Merzbacher disease‐like HLD, movement‐predominant HLD, epileptic HLD, peripheral neuropathy‐associated HLD, and syndromic/systemic HLD phenotypes. Notably, approximately one‐third of individuals with EPRS1‐related disease lacked hypomyelination despite significant neurological involvement. Prominent movement disorders, particularly dystonia and tremor, were frequent in HSPD1‐ and NKX6.2‐related disease. Seizures, often severe or refractory and associated with developmental impairment, were core features in AIMP1‐ and AIMP2‐related disease. Progressive macrocephaly emerged as a consistent feature in individuals with FAM177A1 deficiency.

**Conclusion:** These findings suggest that MRI‐defined HLD classifications do not fully capture the clinical and biological heterogeneity of these disorders. An integrated framework combining clinical subgrouping with imaging and molecular diagnosis may better reflect disease mechanisms, improve diagnostic interpretation, and guide future research.

**Disclosure:** Nothing to disclose.

## LB_04

### Early immune activation in the preclinical phases of immune‐mediated neurological diseases

#### H. Vietzen^1^, R. Reinecke ^2^, J. Nolte^3^, L. Kühner^1^, S. Berger^1^, J. Camp^1^, M. Ponleitner^3^, K. Rostásy^4^, H. Saucke^4^, F. Kauth^4^, G. Koukou^4^, S. Sommer^4^, E. Wendel^4^, M. Graninger^1^, V. Endmayr^2^, K. Koebl‐Shkreli^2^, S. Nitsch^2^, J. Wachutka^2^, E. Waubant^5^, S. Mar^6^, A. Waldman^7^, L. Krupp^8^, C. Casper^9^, T. Schreiner^10^, T. Chitnis^11^, L. Weidner^12^, C. Pistorius^12^, C. Jungbauer^12^, M. Reindl^13^, B. Kornek^3^, M. Breu^3^, G. Bsteh^3^, H. Lassmann^14^, E. Puchhammer‐Stöckl^1^, T. Berger^3^, R. Höftberger^2^, P. Rommer^3^

##### ^1^Center for Virology, Medical University of Vienna, Vienna, Austria, ^2^Division of Neuropathology and Neurochemistry, Department of Neurology, Medical University of Vienna, Vienna, Austria, ^3^Department of Neurology, Medical University of Vienna, Vienna, Austria, ^4^Department of Pediatric Neurology, Children's Hospital Datteln, Witten/Herdecke University, Datteln, Germany, ^5^Department of Neurology, University of California San Francisco, San Francisco, CA, USA, ^6^Washington University School of Medicine in St. Louis, St. Louis, MO, USA, ^7^Children's Hospital of Philadelphia, Philadelphia, PA, USA, ^8^NYU Langone Multiple Sclerosis Comprehensive Care Center, Hassenfeld Children's Hospital at NYU Langone, New York, NY, USA, ^9^Department of Pediatrics, University of Utah School of Medicine, Salt Lake City, UT, USA, ^10^Children's Hospital Colorado, University of Colorado School of Medicine, Aurora, CO, USA, ^11^Massachusetts General Hospital, Harvard Medical School, Boston, MA, USA, ^12^Blood Service for Vienna, Lower Austria and Burgenland, Vienna, Austria, ^13^Clinical Department of Neurology, Medical University of Innsbruck, Innsbruck, Austria, ^14^Center for Brain Research, Medical University of Vienna, Vienna, Austria

**Background and aims:** The temporal relationship between disease‐specific autoantibodies and biomarkers of CNS injury before diagnosis of multiple sclerosis (MS), myelin oligodendrocyte glycoprotein antibody–associated disease (MOGAD), and aquaporin‐4–IgG–positive neuromyelitis optica spectrum disorder (NMOSD) remains unknown. The objective was to elucidate the preclinical trajectory of EBNA‐1–specific, AQP4 as well as MOG antibodies and their association with CNS injury to improve understanding of early pathobiology and biomarker‐based risk stratification.

**Methods:** We performed a multicenter, retrospective, longitudinal case‐control study with pre‐ diagnostic plasma samples from 362 individuals who later developed MS, 145 who later developed MOGAD, and 60 who later developed NMOSD. Plasma IgG levels directed against CNS antigens, MOG, and AQP4, as well as plasma concentrations of neurofilament light chain (pNfL), were quantified, and temporal relationships between immune activation and neuroaxonal injury and clinical disease onset were modeled using linear mixed‐effects models and survival analyses.

**Results:** In MS, EBNA‐1–specific and CNS‐cross‐reactive IgG were elevated up to 77.8 months before diagnosis and preceded first increases in pNfL by mean intervals of 44.9 months, respectively. In NMOSD, AQP4‐IgG seroconversion occurred a mean of 32.5 months before diagnosis and preceded elevations in pNfL by 40.4 months, respectively. In contrast, in MOGAD, elevations in pNfL preceded MOG‐IgG seroconversion by 11.2 months, respectively.

**Conclusion:** In MS and NMOSD, disease‐specific humoral autoimmunity precedes detectable blood biomarkers of CNS injury, whereas in MOGAD, neuroaxonal and astroglial injury becomes evident before circulating MOG‐IgG. These distinct temporal patterns suggest different early pathophysiological mechanisms and may inform early diagnosis and intervention strategies.

**Disclosure:** The study was funded by the Center for Virology (Medical University of Vienna, Vienna), the Department of Neurology (Medical University of Vienna, Vienna), the Austrian Science Fund (FWF): SYNABS, I6565‐B, the Austrian Research Promotion Agency (FFG, project number FO999920011) and the Austrian Society of Neurology. Austria.

## LB_05

### Clinical outcomes and impact of rituximab in CASPR2‐associated disease: a nationwide retrospective study

#### B. Joubert, A. Lebrun, M. Benaiteau, A. Farina, L. Comperat, J. Honnorat

##### CFrench Reference Center for Autoimmune Encephalitis, Hospices Civils de Lyon – MeLiS CNRS UMR 5284 – INSERM U1314, Université Claude Bernard Lyon 1, Lyon, France

**Background and aims:** CASPR2‐associated disease (CASPR2‐ad) is a rare autoimmune neurological disorder characterized by recurrent clinical events and frequent long‐term disability. We aimed to assess the effect of baseline features and treatments on disease course and neurological outcomes.

**Methods:** All patients testing positive for anti‐CASPR2 antibodies at the French Reference Centre for Autoimmune Encephalitis (2011–2025) were retrospectively included. Neurological outcomes, mortality, and clinical event rate were analyzed using generalized linear mixed models, stratified Cox models, and Andersen‐Gill counting‐process models, with treatments as time‐varying covariates.

**Results:** Among 171 patients (89% male; median age 66 years; median follow‐up, 3 years [IQR 1.3–4.8]), the predominant phenotypes were limbic encephalitis (73%), Morvan syndrome (12%), and Isaacs syndrome (9%). Mortality differed markedly across syndromes (*p* < 0.001; Fig. 1A), with thymoma as the only independent predictor (HR 4.59 [1.26–16.73], *p* = 0.021; Fig. 1B). In two‐year survivors, higher baseline mRS and female sex were independently associated with worse neurological outcome (OR 0.19 [0.09–0.39], *p* < 0.001 and OR 0.12 [0.02‐0.71], *p* = 0.02) while Morvan syndrome was associated with higher odds of favorable outcomes (OR 12.06 [1.24‐117.35], *p* = 0.032; AUC 0.868; Fig. 2). Active rituximab therapy was independently associated with a 56% reduction in clinical event rate (HR 0.44 [0.25‐0.77], *p* = 0.004; Fig. 3), with a stronger effect in limbic encephalitis patients (HR 0.35 [0.18–0.66], *p* = 0.001).

**Figure:**
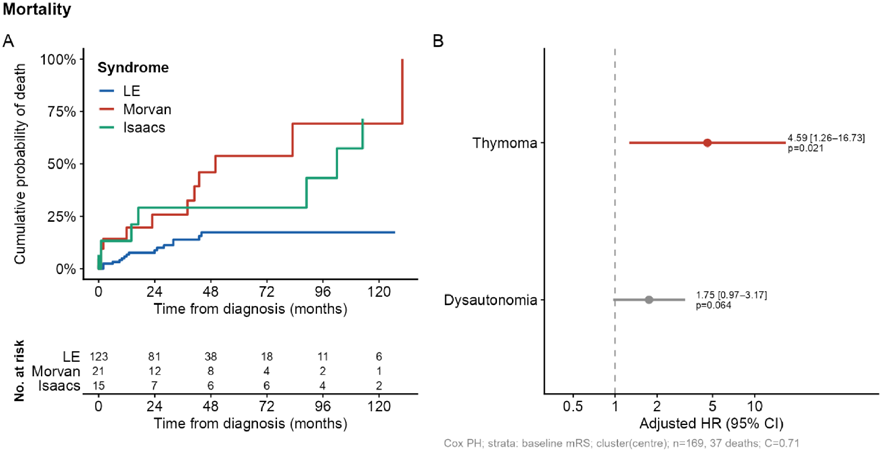
**FIGURE 1** Mortality.

**Figure:**
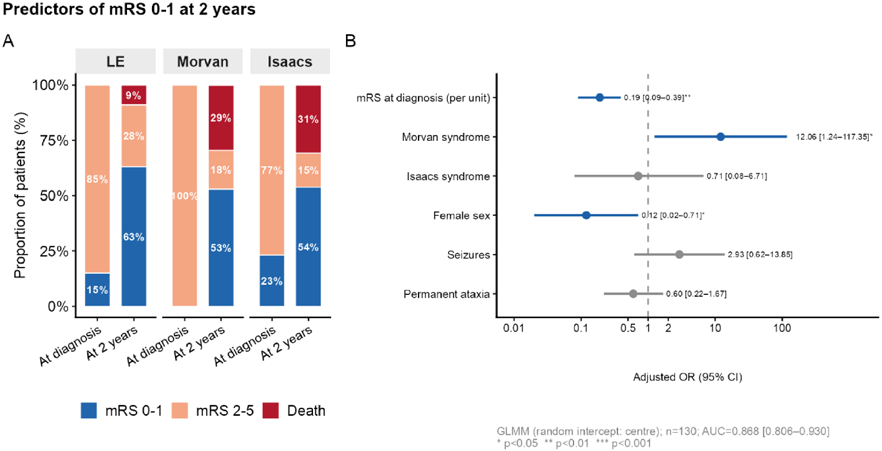
**FIGURE 2** Neurological outcomes at 2 years.

**Figure:**
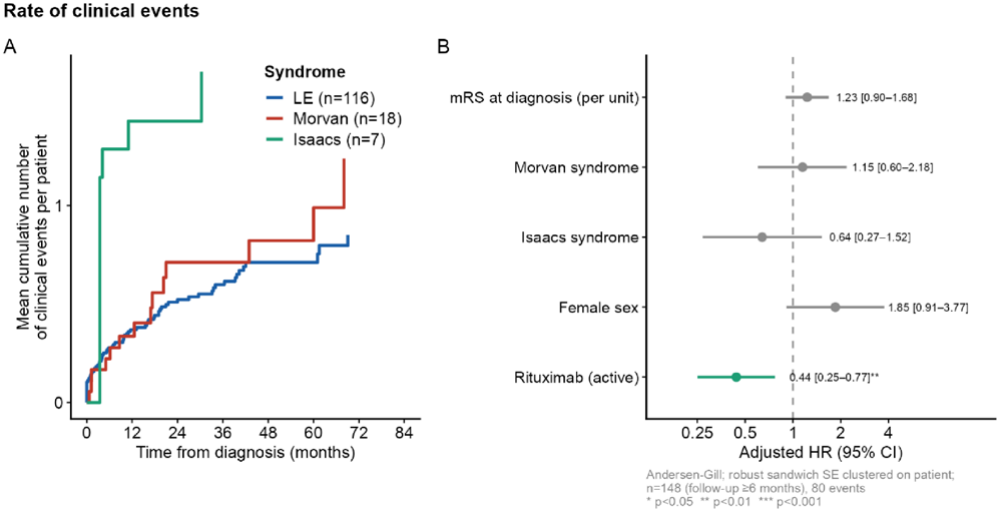
**FIGURE 3** Rate of clinical events (patients with ≥ 6 months follow‐up).

**Conclusion:** Baseline severity and thymoma are the main determinants of neurological outcome and mortality, respectively. Rituximab markedly reduces clinical event rate, supporting its use to control CASPR2‐ad activity. These findings are limited by the retrospective design and potential confounding by indication.

**Disclosure:** This study has been developed within the BETPSY project (ANR‐18‐RHUS‐0012).

## LB_06

### Clemastine as remyelinating treatment in patients with multiple sclerosis and internuclear ophthalmoplegia

#### S. Hof ^1^, D. de Jong^1^, P. Molenaar^1^, J. Twisk^2^, L. van Rijn^3^, B. Uitdehaag^1^, A. Petzold^4^

##### ^1^Neuro‐ophthalmology Expertise Center Amsterdam, Amsterdam UMC location Vrije Universiteit Amsterdam, Neurology, De Boelelaan 1117, Amsterdam, The Netherlands & Amsterdam Neuroscience, Neuroinfection & ‐inflammation, Amsterdam, The Netherlands, ^2^Department of Epidemiology and Data Science, Amsterdam UMC, Amsterdam, The Netherlands, ^3^Neuro‐ophthalmology Expertise Center Amsterdam, Amsterdam UMC location Vrije Universiteit Amsterdam, Ophthalmology, De Boelelaan 1117, Amsterdam, The Netherlands & Onze Lieve Vrouwe Gasthuis, Ophthalmology, Amsterdam, The Netherlands, ^4^The National Hospital for Neurology and Neurosurgery, Moorfields Eye Hospital and the Queen Square Institute of Neurology, UCL, London, United Kingdom

**Background and aims:** Remyelination failure contributes to disability and disease progression in multiple sclerosis (MS). Clemastine has shown promise for therapeutic remyelination in optic neuropathy, but its efficacy in other neural pathways remains uncertain. A key area to explore is the brainstem where lesions cause internuclear ophthalmoplegia (INO) in 25% of people with MS, which can transiently improve with fampridine. We investigated whether clemastine promotes brainstem remyelination and evaluated fampridine response as a remyelination predictor.

**Methods:** In this RCT, 47 people with MS and INO received 4 mg clemastine or placebo twice daily for 6 months. Horizontal saccades conjugacy, a measure of brainstem myelination, was quantified by infrared oculography at baseline, 3, 6, 12, 24 and 36 months. The primary outcome measure was the Versional Dysconjugacy Index (VDI). Before randomisation, participants underwent a 10 mg fampridine challenge. Those with VDI improvement were classified as responders and likely remyelinators. Secondary outcomes included other eye‐movement parameters, OCT, visual acuity, disability, cognition and patient‐reported outcomes.

**Figure:**
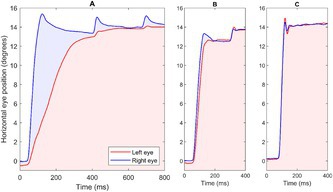
**FIGURE 1** Infrared oculography of rightward saccades: severe INO (A, VDI‐AUC 1.60), mild INO (B, VDI‐AUC 1.26) and no INO (C, VDI‐AUC 0.97). Comparison of shaded area under the curve (VDI‐AUC) quantifies INO severity.

**Results:** The trial ended on 15‐Aug‐2025. Positive fampridine response was observed in 23 subjects (49%), with a balanced distribution between clemastine (*n* = 12) and placebo (*n* = 11) groups (Table 1). Fampridine response was associated with VDI improvement over time (*p* = 0.004). Full treatment efficacy data will be presented at the EAN conference.

**Figure:**
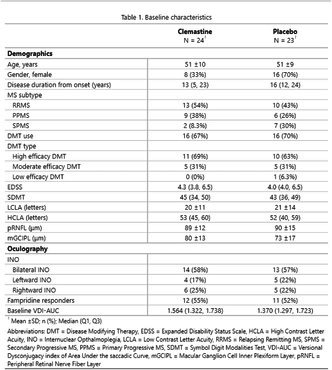
**TABLE 1** Comparison of baseline variables between the clemastine and placebo group in the RESTORE trial.

**Conclusion:** Inclusion of people with INO expands on previous studies on optic neuropathy and provides a platform to study central nervous system remyelination within the brainstem. Selecting for likely remyelinators through fampridine response is feasible and may strengthen future remyelination trials.

**Disclosure:** RESTORE is funded by the VUmc fund foundation (Grant No. 399).

Monday, June 29 2026

Late‐breaking ePoster Session

## LB_07

### GDF‐15 and structural brain changes in community‐dwelling older adults

#### F. Nuti ^1^, M. Valletta^1^, D. Vetrano^1^, E. Laukka^1^, G. Kalpouzos^1^, M. Canevelli^1^, C. Fredolini^3^, G. Bruno^2^, G. Grande^1^

##### ^1^Aging Research Center, Department of Neurobiology, Care Sciences and Society, Karolinska Institutet and Stockholm University, Stockholm, Sweden, ^2^Department of Human Neuroscience, Sapienza University, Rome, Italy, ^3^Affinity Proteomics Stockholm, Science for Life Laboratory, Department of Protein Science, School of Engineering Sciences in Chemistry, Biotechnology and Health (CBH), Royal Institute of Technology (KTH), Solna, Sweden

**Background and aims:** Growth differentiation factor 15 (GDF‐15) is a circulating cytokine reflecting biological aging linked to cognitive decline. Evidence on brain structural correlates is limited. We investigated the associations of blood GDF‐15 with brain volumes and cerebrovascular burden in community‐dwelling older adults free from dementia.

**Methods:** We included 400 community‐dwelling participants from the Swedish National Study on Aging and Care in Kungsholmen (SNAC‐K), free from major baseline cerebrovascular and neurological diseases. GDF‐15 was measured at baseline and analyzed in tertiles. Brain MRI was performed at baseline and after 3 and/or 6 years. Linear mixed models were adjusted for age, sex, education, and chronic comorbidities; secondary analyses further accounted for blood p‐tau217.

**Figure:**
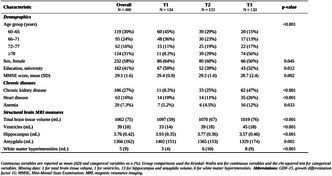
**TABLE 1**. Baseline characteristics of the study population, overall and by GDF‐15 tertiles (T1, T2, T3).

**Results:** Mean age was 70 (SD 9) years; 58% women. Higher GDF‐15 tertiles, adjusted for multiple confounders, showed an association with faster atrophy of total brain tissue (T3 vs T1: β·year = −0.026, 95% CI −0.041, −0.010), hippocampus (β·year = −0.054, 95% CI −0.081, −0.027), and amygdala (β·year = −0.048, 95% CI −0.085, −0.011), and with faster enlargement of the ventricles (β·year = +0.026, 95% CI 0.006, 0.045) and accumulation of white matter hyperintensities (WMH) (β·year = +0.032, 95% CI 0.003, 0.061) over time. Findings were robust to comorbidity adjustment and APOE ε4; associations with total brain tissue, hippocampal, and ventricular trajectories remained significant after adjustment for blood p‐tau217.

**Figure:**
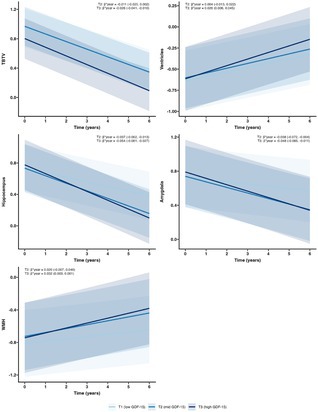
**FIGURE 1** Annual rate of change in volumes of total brain tissue (TBTV), lateral ventricles, hippocampus, amygdala, and white matter hyperintensities (WMH) accumulation over time, by baseline tertiles levels of GDF‐15.

**Figure:**
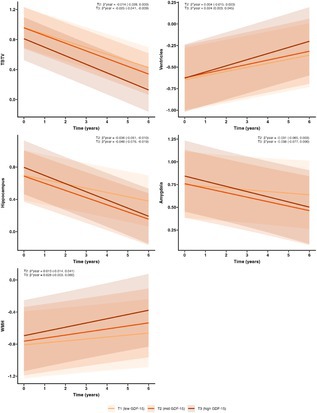
**FIGURE 2** Annual rate of change in volumes of total brain tissue (TBTV), lateral ventricles, hippocampus, amygdala, and white matter hyperintensities (WMH) accumulation over time, by baseline tertiles levels of GDF‐15 adjusted for baseline ptau217 levels.

**Conclusion:** Higher GDF‐15 levels were associated with faster atrophy of total brain tissue, hippocampus, and amygdala, and with ventricular enlargement and WMH over 6 years, supporting GDF‐15 as a biomarker of accelerated age‐related brain atrophy.

**Disclosure:** Nothing to disclose.

## LB_08

### Real‐world clinical outcomes with lecanemab in APOE ε4 heterozygotes and non‐carriers: findings from the LEADER study

#### C. Schreiber ^1^, B. Berry^2^, C. Camargo^3^, T. Chabrashvili^4^, G. Cooper^5^, N. Frost^6^, S. Giles^7^, C. Leahy^8^, M. Rosenbloom^9^, M. Sabbagh^10^, M. Sadowski^11^, P. Schulz^12^, J. Soria^13^, B. Tousi^14^, D. Weisman^15^, F. Frech^16^, C. Adams^16^, D. Jones^16^

##### ^1^Missouri Memory Center, Citizens Memorial Hospital, Bolivar, USA, ^2^Minneapolis Clinic of Neurology, Minneapolis, USA, ^3^Department of Neurology and Evelyn F. McKnight Brain Institute, University of Miami Miller School of Medicine, Miami, USA, ^4^SUNY Upstate Medical University, New York, USA, ^5^Norton Neuroscience Institute, Louisville, USA, ^6^University of Utah Department of Neurology, Salt Lake City, USA, ^7^Memory Treatment Centers, Jacksonville Beach, USA, ^8^Memorial Healthcare Institute for Neuroscience, Owosso, USA, ^9^Memory and Brain Wellness Center and Department of Neurology, University of Washington, Seattle, USA; University of Washington Alzheimer's Disease Research Center, Seattle, USA, ^10^Department of Neurology, Barrow Neurological Institute, St. Joseph's Hospital and Medical Center, Phoenix, USA, ^11^Department of Neurology, NYU Langone Health, New York, USA; Department of Psychiatry, NYU Langone Health, New York, USA; Department of Biochemistry and Molecular Pharmacology, NYU Langone Health, New York, USA, ^12^Neurocognitive Disorders Center, Department of Neurology, The McGovern Medical School of UTHealth‐Houston, Houston, USA, ^13^The Neuron Clinic, Chula Vista, USA; Department of Neurosciences, University of California San Diego, La Jolla, USA, ^14^Cleveland Clinic, Cleveland, USA, ^15^Abington Neurological Associates, Abington, USA, ^16^Eisai Inc, Nutley, USA

**Background and aims:** Real‐world data describing lecanemab clinical and safety outcomes by apolipoprotein E (APOE ε4) status, including amyloid‐related imaging abnormalities (ARIA) incidence, remain limited. This interim analysis from LEADER characterizes clinical outcomes with lecanemab in APOE ε4 heterozygotes and non‐carriers (APOE ε4 homozygotes were excluded to align with the ex‐US label).

**Methods:** LEADER is a multicenter, retrospective, real‐world study conducted across 15 geographically diverse U.S. neurology practices. Sites abstracted deidentified medical chart data for patients who received ≥ 7 biweekly intravenous lecanemab infusions. This descriptive analysis assessed clinician‐reported change in disease stage from baseline to last follow‐up, with improvement defined as a shift from mild Alzheimer's disease dementia to mild cognitive impairment (data cutoff: March 26, 2026).

**Results:** Of 309 patients in LEADER, 256 were included in this analysis (excluding APOE ε4 homozygotes): 155 (50.2%) were heterozygous and 101 (32.7%) were noncarriers. ARIA—edema/effusion (ARIA‐E) occurred in 12 (7.7%) heterozygotes and 7 (6.9%) noncarriers. No intracerebral hemorrhages (> 1 cm) occurred. Mean treatment duration at last follow‐up was 471.3 and 437.6 days, respectively. Over the observation period, 85.2% of heterozygotes were stable or improved (80.0% stable; 5.2% improved) and 87.1% of noncarriers were stable or improved (81.2% stable; 5.9% improved).

**Figure:**
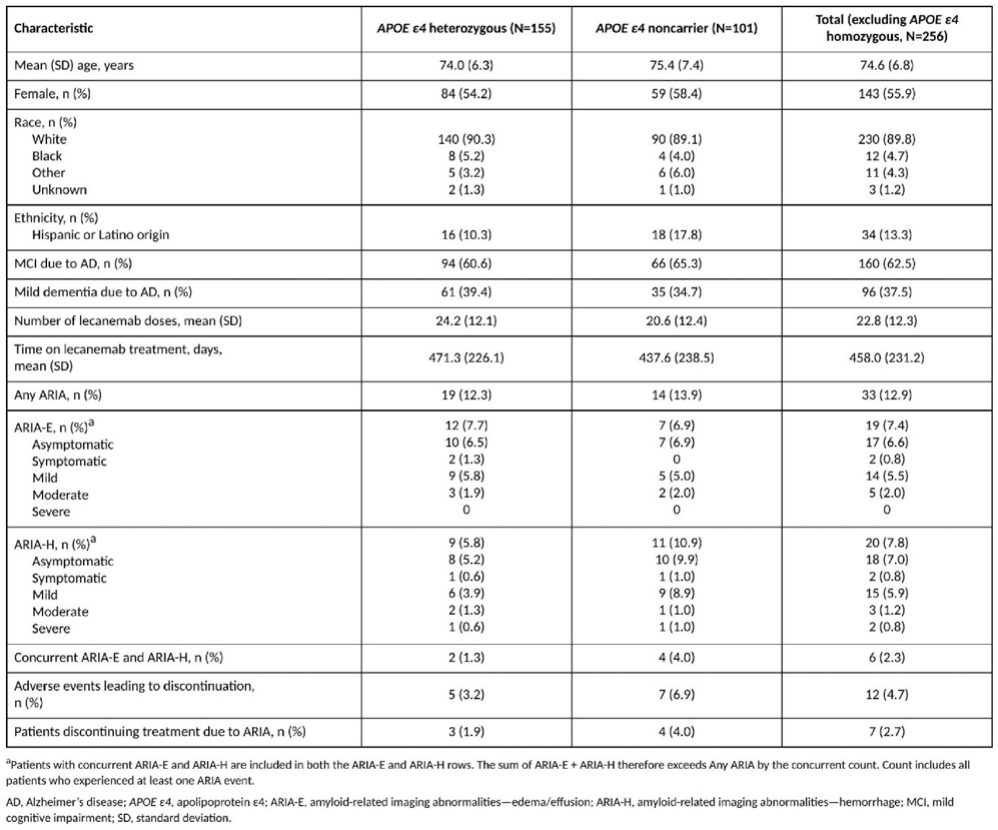
**TABLE 1** Patient characteristics.

**Conclusion:** ARIA‐E occurred in 7.4% of patients and 86.0% were reported as stable or improved over a mean observation period of 458.0 days. Similar outcomes were observed in APOE ε4 heterozygotes and noncarriers. The full data set (data cutoff: May 2026) will further characterize real‐world clinical outcomes including ARIA risk and disease progression across APOE ε4 heterozygotes and noncarriers.

**Disclosure:** BB receives consulting/speaker fees from Eisai Inc., and Eli Lilly and has royalties/licenses for Cognitive Neuroscience Space. CC receives consulting/speaker fees from Eisai Inc. TC receives grant/research support from Karuna Therapeutics and consulting/speaker fees from ALZ‐NET and Eisai Inc. GC receives grant/research support from Eisai Inc., Eli Lilly, Novartis, Davos Alzheimer's Collaborative. SG receives consulting/speaker fees from Eisai Inc., and Eli Lilly. CL receives grant/research support from C2N Diagnostics, Davos Alzheimer's Collaborative, Eisai Inc., Eli Lilly and Neurogen Biomarking, receives consulting/speaker fees from Biogen, C2N Diagnostics, Eisai Inc., Eli Lilly and Neurogen Biomarking. MR receives consulting/speaker fees from Eisai Inc., Eli Lilly and Johnson & Johnson. MSab has stock/stock options in Alzheon and Lighthouse Pharmaceuticals, is on the Board of Directors for CervoMed, and receives consulting/speaker fees from Abbvie, Alzinova, Anavex, Cognito Therapeutics, Eisai Inc., Eli Lilly, GSK, NeuroTherapia, Otsuka, Recall Therapeutics, and Signant. MSad has stock/stock options at Pfizer Inc, receives grant/research support from Alnylam, Biogen Inc., BMS, Eisai Inc., Eli Lilly, GSK, Janssen Pharmaceuticals, Ono Pharmaceuticals, Neurim Pharmaceuticals, and Novo Nordisk, receives consulting/speaker fees from Axsome Therapeutics, Alzheon, Biogen Inc., Cognition Therapeutics, Eisai Inc., Eli Lilly & Co, GSK, Oligomerix, Owkin, Novartis, and has other financial/material support from Fisher Center for Alzheimer's Research Foundation. CS receives consulting/speaker fees from C2N Diagnostics, Eisai Inc., Eli Lilly, and Pfizer and receives research support from Eisai Inc., Eli Lilly, Genetech, Novartis, and Roche. PS receives grant/research support from NIH, receives consulting/speaker fees for Acadia, Eisai Inc., and Eli Lilly, and is on the Board of Directors for the Alzheimer's Association of Houston and the FTLDA of San Antonio. JS receives grant/research support from Alzheimer's Association, Annovis Bio, Aribio, Biogen, Eisai Inc., Eli Lilly, Karuna/BMS, NIH‐NIA, and Novartis and consulting/speaker fees from Arrowhead Pharmaceuticals, Biogen, Eisai Inc., Eli Lilly, Genetech, Global Learning Collaborative and Merck. BT receives grant/research support from Acadia, Biogen, Cognition Therapeutics, EIP, Eisai Inc., NIH‐NIA, and Novo Nordisk, receives consulting/speaker fees for Biogen, Eisai Inc., Lundbeck, Novo Nordisk, and Sunbird Bio. DW receives grant/research support from Acadia, Acumen, Alnylam, Alzheimer's Association, Cereval, Cognition, Eisai Inc., Eli Lilly, Novartis, Sage, Sanofi, Serono, and Roche and receives consulting/speaker fees from American Stroke Association, Biogen, Eisai Inc., Lanheus and Novartis. CA, FF, DRJ are employees of Eisai Inc. NF reports no conflicts of interest.

## LB_09

### Bocunebart (anti‐PACAP monoclonal antibody Lu AG09222) in adults with migraine and prior preventive treatment failures: Phase 2 program outcomes

#### J. Ailani^1^, R. Phul^2^, I. Florea^2^, M. Josiassen^2^, S. Schmidt^2^A. Blumenfeld^3^, M. Ashina ^4,5^

##### ^1^Department of Neurology, Medstar Georgetown University Hospital, Headache Center, Washington, DC, USA, ^2^H. Lundbeck A/S, Valby, Copenhagen, Denmark, ^3^The Los Angeles Headache Center, Los Angeles, CA, USA, ^4^Department of Clinical Medicine, Faculty of Health and Medical Sciences, University of Copenhagen, Copenhagen, Denmark, ^5^Department of Neurology, Center for Discoveries in Migraine, Danish Headache Center, Copenhagen University Hospital – Rigshospitalet, Copenhagen, Denmark

**Background and aims:** Pituitary adenylate cyclase‐activating polypeptide (PACAP) is a novel therapeutic target in migraine prevention. We assessed the efficacy and safety of bocunebart (Lu AG09222), an anti‐PACAP monoclonal antibody (mAb), in adults with migraine for whom prior preventive treatments have failed. We report outcomes from the PROCEED trial and the phase 2 program.

**Methods:** The phase 2 program comprised the proof‐of‐concept phase 2a HOPE trial (NCT05133323) and the adaptive phase 2b PROCEED trial (NCT06323928), evaluating dose and route of administration. The PROCEED primary endpoint was change from baseline in monthly migraine days (MMDs) over Weeks 1–12. Efficacy in participants with chronic migraine (CM) was evaluated in pooled post‐hoc analyses across all doses and parts of PROCEED+HOPE.

**Results:** For PROCEED (intravenous part), the primary endpoint was met; a statistically significant reduction in MMDs was observed with bocunebart dose A versus placebo over Weeks 1–12 (−4.24 [standard error 0.63] vs −2.86 [0.53]; *p* = 0.0178 [2‐sided]). Over Weeks 9–12, in the pooled CM subgroup (PROCEED+HOPE), pooled bocunebart doses demonstrated greater improvements from baseline versus placebo in MMDs (−5.94, [0.42] vs −3.63 [0.53]; *p* < 0.001), monthly headache days (−6.90, [0.42] vs −4.32 [0.54]; *p* < 0.001) and MMDs with acute medication use (−5.21, [0.39] vs −3.53 [0.50]; *p* < 0.05). Bocunebart was generally well tolerated; no new safety signals were identified.

**Conclusion:** Positive phase 2 findings support PACAP inhibition as a novel approach in migraine prevention. Overall, bocunebart reduced migraine burden in a severely impacted population with high unmet need. These findings support bocunebart's continued development as a potential first‐in‐class anti‐PACAP mAb.

**Disclosure:** Jessica Ailani reports honoraria from AbbVie, Axsome Therapeutics, Amneal Pharmaceuticals, Aspeya, Bausch, Eli Lilly, Lundbeck, Ipsen, Merz, Pfizer, Kallyope, and Satsuma; institutional grants for clinical trials from Ipsen, Lundbeck, Pfizer, Merz, and ShiraTronics; grants paid to the principal investigator for clinical trials from Mi‐Helper; and stock options from MINDED; Ravinder Phul, Ioana Florea, Mette Krog Josiassen, and Simon Nitschky Schmidt are employees of H. Lundbeck A/S; Messoud Ashina reports personal fees for consulting, advisory board participation, and speaker honoraria from AbbVie, AstraZeneca, Eli Lilly, GlaxoSmithKline, Incyte, Lundbeck, Novartis, Pfizer, and Teva Pharmaceuticals; the clinical trials summarized in this abstract were funded by H. Lundbeck A/S.

## LB_10

### ROSSINI 12‐month interim results: Safety and effectiveness of 24‐hour Foslevodopa/Foscarbidopa in Parkinson's disease

#### W. Jost^1^, F. Bergquist^2^, A. Evans^3^, S. Hassin‐Baer^4^, R. Hauser^5^, T. Henriksen^6^, I. Malaty^7^, T. Mestre^8^, P. Mir^9^, R. Rodriguez^10^, P. Schwingenschuh^11^, M. Simu^12^, L. Bergmann ^13^, S. Pan^13^, S. Caughlin^13^, M. Gopalkrishnan^13^, P. Kukreja^13^, M. O'Meara^13^, J. Carlos Parra^13^, M. Shah^13^, J. Aldred^14^

##### ^1^Parkinson‐Klinik Ortenau, Wolfach, Germany, ^2^Department of Clinical Neuroscience, Institute of Neuroscience and Physiology, University of Gothenburg, Gothenburg, Sweden; Department of Neurology, Sahlgrenska University Hospital, Gothenburg, Sweden, ^3^The Royal Melbourne Hospital, Parkville, Victoria, Australia, ^4^Movement Disorders Institute, Department of Neurology, Sheba Medical Center, Tel‐Hashomer, Israel; Gray Faculty of Medical and Health Sciences, Tel Aviv University, Tel Aviv, Israel, ^5^University of South Florida, Tampa, FL, USA, ^6^Movement Disorder Clinic, University Hospital of Bispebjerg, Copenhagen, Denmark, ^7^Department of Neurology, Fixel Institute for Neurological Diseases, University of Florida, Gainesville, FL, USA, ^8^Division of Neurology, Department of Medicine, The Ottawa Hospital, Ottawa Hospital Research Institute, University of Ottawa, Ottawa, Canada, ^9^Unidad de Trastornos del Movimiento, Servicio de Neurología, Instituto de Biomedicina de Sevilla, IBiS/Hospital Universitario Virgen del Rocío/CSIC/Universidad de Sevilla, Seville, Spain, ^10^Neurology One, University of Central Florida, Orlando, FL, USA, ^11^Department of Neurology, Medical University of Graz, Graz, Austria, ^12^Department of Neurology, Victor Babes University of Medicine and Pharmacy, Timisoara, Romania, ^13^AbbVie Inc., North Chicago, IL, USA, ^14^Selkirk Neurology & Inland Northwest Research, Spokane, WA, USA

**Background and aims:** Foslevodopa/foscarbidopa (LDp/CDp) is a nonsurgical 24‐hour/day continuous subcutaneous infusion for patients with advanced Parkinson's disease (aPD) whose motor fluctuations are uncontrolled on oral medications. Twelve‐month safety and effectiveness data from routine clinical practice were captured.

**Methods:** ROSSINI (NCT06107426) is an ongoing 3‐year multicountry, prospective, observational study of adults with aPD who are LDp/CDp‐naïve (cohort A) or transitioning from LDp/CDp open‐label extension studies (NCT04379050/NCT04750226, cohort B). Second interim results for cohort A patients (N = 202) enrolled ≥ 12 months (M) before 16December2025 are presented. Adjusted mixed‐effects models for repeated measurements (continuous outcomes) were utilized.

**Results:** At baseline, patients had a mean (SD) age of 68.1 y (9.1 y), PD duration of 12.1 y (5.7 y), and OFF time of 4.8h (2.6 h, Table 1). From initial prescription to M12, mean daily LD equivalent dose during infusion (1414.7 mg–1471.9 mg) and LDp/CDp base infusion rates (58.9 mg/h LD–67.1 mg/h LD) moderately increased; 21/201 (10.4%) patients received LDp/CDp as a monotherapy until final visit/M12. Patients on LDp/CDp showed statistically significant sustained reductions in motor fluctuations and nonmotor symptoms by M12, including in King's Parkinson's Disease Pain Scale scores (−8.4; Figure 1, Table 2). Patients < 65 y of age and < 10 y PD duration (*n* = 22) showed numerically larger reductions in 39‐item Parkinson's Disease Questionnaire scores than the overall population (Figure 1). For safety, 149/201 (74.1%) patients reported ≥ 1 adverse event, mostly nonserious and mild‐moderate, with hallucinations and infusion site events most frequent (Table 1).

**Figure:**
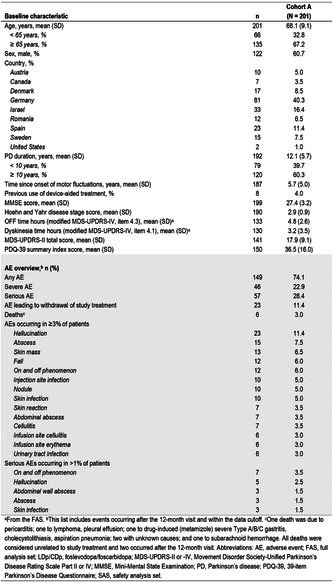
**TABLE 1** Baseline characteristics and overview of adverse events through 12 months of LDp/CDp treatment in the second interim analysis population (SAS).

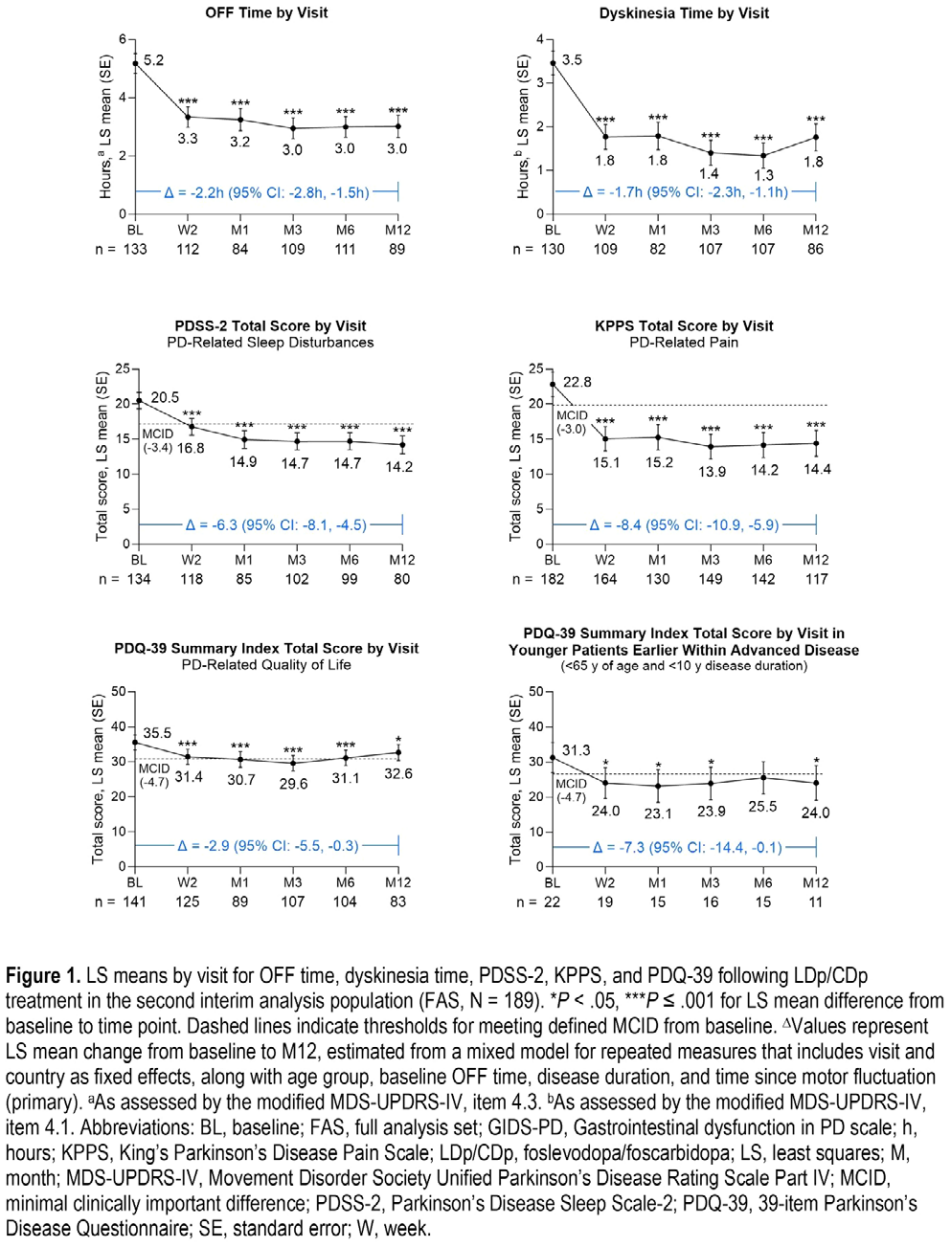

**Figure:**
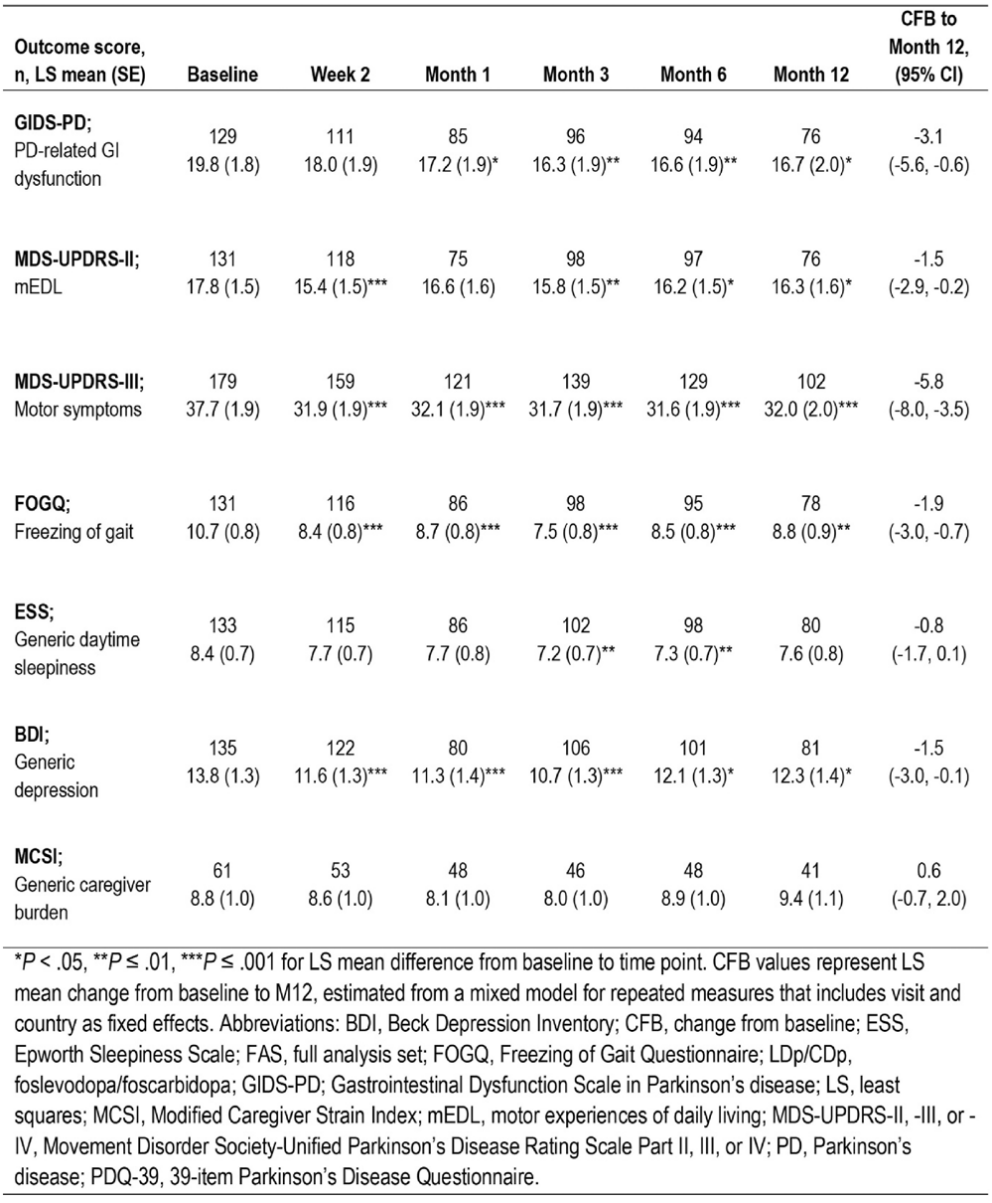
**TABLE 2** LS mean by visit and change from baseline to month 12 for additional effectiveness outcomes among patients on LDp/CDp treatment in the second interim analysis population (FAS, N 0 189).

**Conclusion:** ROSSINI interim results demonstrate sustained reductions in PD symptoms following 12M of LDp/CDp treatment with an acceptable safety profile.

**Disclosure:** WHJ: AbbVie, Bial, Desitin, Stada, UCB, and Zambon. FB: AbbVie, Global Kinetics Corporation. AE: Abbott, AbbVie, Encapsulate, Ipsen, Michael J. Fox Foundation, National Health and Medical Research Council, Pfizer, Seqirus, Stada. SHB: AbbVie, Allergan, Medison, Neuroderm, Takeda, Teva. RAH: AbbVie, ABLi Therapeutics, Amneal, Annovis Bio, Axial, Biogen, Cavion, Cerevance, Cerevel, Clario, Enterin, F. Hoffman La Roche Ltd, Forsee, Genentech, Global Kinetics Corporation, HanAll Biopharma, Inhibikase, Intrance (PSG), Kiefe RX, Kyowa Kirin, Michael J. Fox Foundation, Mitsubishi Tanabe, Motric Bio, Nano PharmaSolutions, Neurocrine Biosciences, NeuroDerm, NDP, Parkinson's Foundation, Photopharmics, Regenxbio, Revance, Sage, Scion Neurotim, Serina Supernus, Stoparkinson, Sumitomo, Sun Pharma Advanced Research Company, Supernus, Teva, Theravance, TrueBinding, UCB, Zambon. TH: AbbVie, Britannia, Convatec, Ipsen, Lundbeck, M15‐741/M15‐737 studies, NeuroDerm, Nordic Infucare. IAM: AbbVie, Aevum, Dystonia Coalition, Emalex, Medscape, NeuroDerm, Parkinson's Foundation, Parkinson Study Group, Praxis, Revance, Robert Rose Publishers, Sage. TM: AbbVie, Biogen, CHDI, Canadian Institutes of Health Research, Michael J Fox Foundation, nQ Medical, Ontario Research Fund, Parkinson Canada, Roche, Sunovion, University of Ottawa/Parkinson Research Consortium, Weston Brain Foundation, Valeo. PM: Abbott, AbbVie, Allergan, Bial, Britannia, Consejería de Economía, Innovación, Ciencia y Empleo de la Junta de Andalucía, Consejería de Salud y Bienestar Social de la Junta de Andalucía, Consejería de Transformación Económica, Industria, Conocimiento y Universidades de la Junta de Andalucía, Instituto de Salud Carlos III‐Fondo Europeo de Desarrollo Regional, Italfarmaco, Merz, Spanish Ministry of Science and Innovation, Teva, UCB, Zambon. RR AbbVie, ACR, Biogen, Cerevel, Cerevance, CND Life Sciences, Eisai, Eli Lilly, Koneksa, Kyowa Kirin, MTPA, NeuroDerm, Neurocrine, Teva. PS: AbbVie, Bial, Boston Scientific, Britannia, FWF Austrian Science Fund, MDS Clinical Neurophysiology Study Group, MDS Tremor Study Group, Merz, Roche, Stada, Takeda. MS: AbbVie, AOP Orphan, BI, KRKA, Merck, Novartis, Roche, Sanofi, Servier, Teva, UCB. LB, SP, SC, MG, PK, MO, JCP, MBS: AbbVie. JA: Abbott, AbbVie, AC Immune, Allergan (now AbbVie), Annovis, Aptinyx, AstraZeneca, Atara, Athira, Biogen, Biovie, Boston Scientific, Celgene, Cerevance, Cerevel, Denali, EIP, Eli Lilly, Impax, Inhibikase, IRL Therapeutics, Medtronic, Merz, Neuraly, Neurocrine, Neuoderm, Novartis, PD Gene/PSG, Praxis, Revance, Roche/Genentech, Sage, Sanofi/Genzyme, Scion Neurostem, Takeda, Teva, Theravance, Triplet/HSG, UCB, US World Meds.

## LB_11

### Efficacy and safety of Efgartigimod PH20 SC in adults with ocular myasthenia gravis: ADAPT OCULUS interim results

#### K. Claeys ^1^, V. Juel^2^, C. Barnett‐Tapia^3^, A. Meisel^4^, J. Howard Jr^5^, C. Zhao^6^, J. Xi^6^, A. Uzawa^7^, S. Reddel^8^, C. Antozzi^9^, Ł. Rzepiński^10^, R. Álvarez‐Velasco^11^, O. Lukash^12^, R. Jimenez^12^, F. Gistelinck^12^, F. Verhamme^12^, S. Wong^13^

##### ^1^Department of Neurology, University Hospitals Leuven, Leuven, Belgium; Laboratory for Muscle Diseases and Neuropathies, KU Leuven, Leuven, Belgium, ^2^Department of Neurology, Duke University School of Medicine, Durham, North Carolina, USA, ^3^Division of Neurology, University of Toronto, Toronto, Ontario, Canada, ^4^Department of Neurology and Neuroscience Clinical Research Center, Charité – Universitätsmedizin Berlin, Berlin, Germany, ^5^Department of Neurology, The University of North Carolina, Chapel Hill, North Carolina, USA, ^6^Department of Neurology, Huashan Hospital, Fudan University, Shanghai, China, ^7^Graduate School of Medicine, Chiba University, Chiba, Japan, ^8^Department of Neurology, Concord Hospital, Sydney, New South Wales, Australia, ^9^Neuroimmunology and Neuromuscular Diseases Unit, Fondazione IRCCS Istituto Neurologico Carlo Besta, Milan, Italy, ^10^Department of Neurology, 10th Military Research Hospital and Polyclinic, Bydgoszcz, Poland; Department of Clinical Medicine, Faculty of Medicine, University of Science and Technology, Bydgoszcz, Poland, ^11^Department of Neurology, Hospital Universitario Ramón y Cajal, Madrid, Spain, ^12^argenx, Ghent, Belgium, ^13^Moorfields Eye Hospital, London, UK

**Background and aims:** There is an unmet need for approved and effective treatments for patients with ocular myasthenia gravis (oMG). Subcutaneous (SC) efgartigimod PH20 is a human immunoglobulin G1 (IgG1) antibody Fc fragment coformulated with recombinant human hyaluronidase PH20 that selectively reduces IgG levels by blocking neonatal Fc receptor–mediated IgG recycling. Retrospective data analyses supporting the approval of efgartigimod for treatment of generalized MG indicated improvements in ocular symptoms. Here, we present interim results from the Phase 3 ADAPT OCULUS trial (NCT06558279) evaluating the efficacy and safety of efgartigimod PH20 SC in adults with oMG.

**Methods:** Adults with oMG (MGFA Class I) and Myasthenia Gravis Impairment Index (patient‐reported outcome; MGII [PRO]) ocular score ≥ 6 were randomized 1:1 to receive 4 once‐weekly efgartigimod PH20 SC 1000 mg or placebo injections via prefilled syringe, with 4 weeks of follow‐up. Participants could continue to Part B, a ≤ 2‐year open‐label period.

**Results:** The primary endpoint was met with statistically significant (*p* = 0.012) improvement from baseline in MGII (PRO) ocular score at Week 4 with efgartigimod compared with placebo. Least squares mean change from baseline to Week 4 in MGII (PRO) ocular score was −4.04 points with efgartigimod versus −1.99 for placebo. A reduction in all ocular symptoms was reported, and no new safety concerns were identified.

**Conclusion:** ADAPT OCULUS is the first registrational trial designed specifically to evaluate a targeted therapy for patients with oMG. These results support the efficacy and safety of efgartigimod PH20 SC treatment across multiple cycles in participants with oMG.

**Disclosure:** VCJ, CZ, and JX have nothing to disclose. OL, RHJ, FG, and FMV are employees of argenx. CB‐T, AM, JFHJ, AU, SWR, KGC, CA, ŁR, RÁ‐V, and SHW have reported financial/nonfinancial relationships with argenx at the time of submission

## LB_12

### Neuronal apoptosis redirects disease‐associated microglial states in presymptomatic Alzheimer's iPSC models

#### A. Dapkute ^1^, K. Lee^2^, D. Agarwal^3^, M. Cader^1^

##### ^1^Nuffield Department of Clinical Neurosciences, University of Oxford, UK, ^2^Nuffield Department of Medicine, Centre for Human Genetics, University of Oxford, UK, ^3^Kennedy Institute of Rheumatology, University of Oxford, UK

**Background and aims:** This study investigated how microglia from presymptomatic AD patients respond to an AD‐like neuronal environment using patient‐derived induced pluripotent stem cell (iPSC) models.

**Methods:** Twenty iPSC lines from presymptomatic AD participants from the Deep and Frequent Phenotyping study were differentiated into microglia. iPSC‐microglia were exposed to non‐apoptotic (NAN) or apoptotic neurons (AN) and analysed by scRNA‐sequencing. Following quality control, 14 lines (11 APOE3/E4, 3 APOE4/E4) were retained. Data were preprocessed using Panpipes and annotated using a published microglia atlas. Clustering and batch correction were performed with Monocle3, while trajectory inference and pseudotime‐associated gene analysis were performed using Slingshot and tradeSeq. Differential expression and gene ontology enrichment analyses were conducted using Seurat and ClusterProfiler.

**Results:** Trajectory analysis identified two trajectories leading from cycling to disease‐associated microglia (DAM). Trajectory towards GPNMB‐high CXCR4+ TREM2+ Lipox‐DAM via TREM2+ IFN responsive microglia was preferred in NAN environment. Alternatively, when subjected to AN, trajectory towards LPL‐high TREM2+ DAM via MITO‐high DAM was more prominent. Comparing microglia subtype ratios between NAN and AN environments showed a decrease in TREM2+ IFN responsive microglia (14.2% in NAN to 1.0% in AN) and an increase in MITO‐high DAM (7.2% in NAN to 15.3% in AN). Compared with TREM2+ IFN‐responsive microglia, MITO‐high DAM showed enrichment of endocytosis‐related pathways and reduced immune‐response signatures.

**Conclusion:** Our findings suggest that neuronal apoptosis redirects presymptomatic AD microglia toward a metabolically active, endocytosis‐associated DAM state while reducing IFN‐responsive immune programs. These results raise the possibility that AD‐related neuronal damage may impair protective microglial immune responses early in disease pathogenesis.

**Disclosure:** Nothing to disclose.

## LB_13

### How did Pyridostigmine gain its reputation with POTS? A systematic review and head‐to‐head intervention meta‐analysis

#### L. Überall, A. Pavelic, W. Struhal

##### Department of Neurology, University Hospital Tulln, Tulln, Austria

**Background and aims:** Pharmacotherapy of postural orthostatic tachycardia syndrome (POTS) commonly includes beta‐blockers, ivabradine, midodrine, fludrocortisone and pyridostigmine. However, the use of pyridostigmine in POTS is still largely based on limited evidence.

To perform a systematic review and head‐to‐head intervention meta‐analysis of studies evaluating the efficacy of pyridostigmine in reduction of heart rate in standardized autonomic function testing in comparison to beta‐blockers, ivabradine and midodrine.

**Methods:** We searched PubMed, Embase, CINAHL as well as Cochrane Library for results up to May 25th, 2026, and evaluated full text articles for 38 of 443 search results. Six articles met all selection criteria. The respective heart rate change results (before and after intervention) of these 6 articles were pooled together and analyzed in a head‐to‐head manner (Pyridostigmine vs. beta‐blockers and Pyridostigmine vs. ivabradine). All analyses were carried out using Review Manager 5.4.

**Results:** In the 6 articles, pyridostigmine showed no superiority in comparison to beta‐blockers or ivabradine. The standardized mean difference (SMD) was −10.38 (95% confidence interval (CI) –47.90 to 27.14) for pyridostigmine vs. beta‐blockers, and −3.63 (CI –56.54 to 49.27) for pyridostigmine vs. ivabradine.

**Conclusion:** Based on the currently available literature, pyridostigmine should not be considered a first‐line therapy in patients with POTS. Our head‐to‐head meta‐analysis and systematic review showed that the available literature is highly heterogeneous and published studies reported diverse primary endpoints. Our findings highlight the need for unanimous and standardized reporting of results (including symptomatologic questionnaires) to accurately evaluate the success of POTS pharmacological interventions.

**Disclosure:** Nothing to disclose.

## LB_14

### AI‐Augmented EEG interpretation: Unlocking timely diagnosis for new‐onset epilepsy patients

#### E. Ménétré ^1^, S. Rayatdoost^1^, A. Lage^1^, M. Seeck^2^, S. Gallotto^1^

##### ^1^dEEGtal Insights SA, Geneva, Switzerland, ^2^EEG and Epilepsy Unit, University Hospitals and Faculty of Medicine, Geneva, Switzerland

**Background and aims:** Approximately five million individuals are diagnosed with epilepsy each year, while many others present with uncertain first‐seizure events. Early diagnosis is essential because timely treatment improves outcomes, yet delays are common due to limited access to electroencephalography (EEG) expertise. Interictal epileptiform discharges (IEDs), the only routinely used EEG biomarker, are highly specific but have low sensitivity. This study aimed to develop and validate an artificial intelligence (AI)‐based diagnostic classifier using resting‐state EEG and routinely available clinical variables to improve early differentiation between epileptic and non‐epileptic first‐seizure presentations.

**Methods:** A transformer‐based model was trained on approximately 10‐minute resting‐state EEG recordings from a real‐world cohort. Patients were classified as having epilepsy or non‐epileptic events after a standardized 2‐year follow‐up. Model inputs included EEG features, expert‐marked IEDs, and clinical variables (age, sex, lesion status). A subject‐level 5‐fold cross‐validation framework was used, with performance assessed using the area under the receiver operating characteristic curve (AUROC), sensitivity, specificity, and accuracy.

**Results:** The cohort included 1248 patients (621 epilepsy; 627 non‐epilepsy). EEG alone yielded an AUROC of 0.72, decreasing to 0.67 in patients without IEDs or epileptogenic lesions. Adding clinical variables improved performance to 0.79. A fusion model combining EEG, IEDs, and clinical data achieved the highest performance (AUROC = 0.82; accuracy = 0.74; specificity = 0.86; sensitivity = 0.61).

**Conclusion:** AI‐derived resting‐state EEG in combination with routinely available clinical variables provides diagnostic value beyond conventional interpretation and may reduce diagnostic uncertainty and treatment delays after a first seizure.

**Disclosure:** This research was co‐financed through a combination of public and private funding sources. Some authors are shareholders and/or full‐time employees of dEEGtal Insights SA.

## LB_15

### Long‐term safety and tolerability of del‐desiran in myotonic dystrophy type 1: Final results from MARINA‐OLE™

#### N. Johnson^1^, J. Hamel^2^, S. Subramony^3^, P. Soltanzadeh^4^, J. Statland^5^, M. Freimer^6^, D. Quan^7^, S. Vaziri^8^, B. McEvoy^8^, M. Fowler^8^, E. Ackermann^8^, A. Davidson^8^, D. Schmitt ^9^

##### ^1^Virginia Commonwealth University, Richmond, USA, ^2^University of Rochester Medical Center, Rochester, USA, ^3^University of Florida, Gainesville, USA, ^4^University of California, Los Angeles, Los Angeles, USA, ^5^University of Kansas Medical Center, Kansas City, USA, ^6^The Ohio State University, Columbus, USA, ^7^University of Colorado Anschutz, Aurora, USA, ^8^Avidity Biosciences, A Novartis Company, San Diego, USA, ^9^Avidity Biosciences, A Novartis Company, Non‐author Presenter, San Diego, USA

**Background and aims:** Myotonic dystrophy type 1 (DM1) is a rare, progressive neuromuscular disorder caused by DMPK mutations, leading to toxic mRNA accumulation, muscle dysfunction, and reduced life expectancy. Delpacibart etedesiran (del‐desiran; AOC 1001) is an investigational antibody oligonucleotide conjugate comprised of a monoclonal antibody targeting transferrin receptor 1 for muscle delivery and an siRNA that induces degradation of DMPK mRNA to treat the underlying cause of DM1.

**Methods:** MARINA‐OLE™ was a phase 2, open‐label extension study assessing long‐term safety and tolerability of del‐desiran in adults with DM1, with follow‐up extending to 37 months. Participants received del‐desiran at either 2 or 4 mg/kg every 13 weeks and later transitioned to 4 mg/kg every 8 weeks, consistent with the phase 3 trial regimen. All participants completed approximately 1 year on this regimen.

**Results:** 37 participants received del‐desiran, 101.3 patient‐years of cumulative exposure with 506 infusions. Most treatment‐emergent adverse events (TEAEs) were mild/moderate. No severe or serious TEAEs were treatment‐related, and none led to treatment discontinuation or death. Laboratory values, including hematology parameters, were stable over time.

**Conclusion:** Del‐desiran showed favorable long‐term safety and tolerability in adults with DM1. These data represent the longest safety results for a DMPK‐targeted therapy and highlight potential of del‐desiran to drive a new treatment paradigm for DM1.

**Disclosure:** Funding: This study was supported by Avidity Biosciences, A Novartis Company.

NEJ has received grant funding from NINDS (R01NS104010, U01NS124974), NCATS (R21TR003184), CDC (U01DD001242), and the FDA (2R01FD006071); receives royalties from the CCMDHI and the CMTHI, and research funds from Avidity Biosciences, A Novartis Company, Takeda, Sanofi Genzyme, Dyne, Novartis, Vertex Pharmaceuticals, Fulcrum Therapeutics, ML Bio, and Sarepta; provided consultation for Arthex, Novartis, AMO Pharma, Takeda.

JH served on Clinical Advisory Committees for Dyne Therapeutics and Vertex Pharmaceuticals; provided disease specific information to Design Therapeutics; as a Q&A panel member for Avidity Biosciences, A Novartis Company, at the MDA Conference; and as the overall Principal Investigator for the PepGen Freedom DM1 Study.

SHS has participated in Advisory Boards for Amicus Therapeutics, Inc., Fulcrum Therapeutics, PTC Therapeutics, Inc., and Reata; is a Principal Investigator for clinical trials by Avidity Biosciences, A Novartis Company, Biogen, Inc., Biohaven Pharmaceuticals, Inc., Fulcrum Therapeutics, Harmony Biosciences, PTC Therapeutics, Inc., Reata, Reneo, and Retrotope; and has discussed forthcoming trials with Solid Biosciences.

PS has a Research Contract Grant through UCLA to perform del‐desiran clinical trials with Avidity Biosciences, A Novartis Company.

JS has served as a Consultant on trial design (Armatus Bio and Epic Bio), Advisory Boards (Avidity Biosciences, A Novartis Company, Dyne, F. Hoffmann‐La Roche, Fulcrum Therapeutics, and Vertex Pharmaceuticals), and for Kate Therapeutics; received funding for clinical studies from Avidity Biosciences, A Novartis Company, Dyne, F. Hoffman‐La Roche, FSHD Canada, and NINDS; received a grant for Muscular Dystrophy Clinical Research Network from MDA; and receives options as part of a non‐employee compensation plan from Dyne.

MF received research support from Alnylam, Argenx, Catalyst Pharmaceuticals, Fulcrum Therapeutics, Momenta Pharmaceuticals, Inc., and UCB, Inc.; has been provided consulting fees by Argenx, Johnson & Johnson, and UCB Inc. for participation in Advisory Boards and by UCB Inc. for serving as a faculty member for speakers bureau training; and has served as Principal Investigator in clinical trials sponsored by Avidity Biosciences, A Novartis Company.

DQ receives clinical research support from Alynlam, Bridge Bio, Momenta, and Avidity; serves on a data safety monitoring board for Argenx; and has received payment from Astra Zeneca for a speaking engagement.

SV, AD, BM, MF, and EJA are employees of Avidity Biosciences, A Novartis Company.

## LB_16

### Safety and efficacy of SR750 in patients with trigeminal neuralgia: A double‐blind, randomized, placebo‐controlled, phase 2 study

#### B. Fan^1^, Q. Han^2^, L. Jin^2^, X. Gong^2^, F. Wang^2^, C. Fei^2^, L. Wang^2^, Y. Chen ^2^, S. Li^2^

##### ^1^Department of Pain Management, China‐Japan Friendship Hospital, Beijing, China, ^2^Shanghai SIMR Biotechnology Co., Ltd, Shanghai, China

**Background and aims:** Trigeminal neuralgia (TN) is a severely disabling disorder featuring sudden, recurrent, electric shock‐like facial pain in the trigeminal nerve distribution. SR750, a first‐in‐class alpha5‐GABAA receptors negative allosteric modulator (NAM) developed for neuropathic pain, has the potential to improve the condition of TN. This study assessed the efficacy and safety of SR750 in Chinese patients with TN.

**Methods:** A phase 2 randomized, double‐blind and placebo‐controlled study (NCT06571448) was initiated in July 2024. Patients diagnosed with primary TN were recruited and randomized into three arms at 1:1:1 ratio to receive SR750 50 mg, 150 mg or placebo twice a day (BID) for 6 weeks. The primary endpoint was change from baseline at week 6 in the weekly average of the daily average pain score (DAPS) of paroxysms using pain intensity numerical rating scale.

**Figure:**
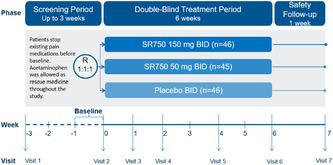
**FIGURE 1** Study design.

**Results:** A total of 138 patients were enrolled, with 45 patients treated with SR750 50 mg BID, 47 treated with SR750 150 mg BID, and 46 treated with placebo. For the primary endpoint, the least square mean (LSM) change from baseline were −0.89, −1.83, and −1.97 in the placebo, SR750 50 mg BID and SR750 150 mg BID groups, respectively. Both SR750 treatment groups were superior to placebo, with between‐group LSM differences of −0.94 (90% CI −1.66, −0.23, *p* = 0.031) and −1.08 (90% CI −1.78, −0.37, *p* = 0.012), respectively.

**Conclusion:** Overall, SR750 low dose and high dose demonstrated statistically and clinically significant efficacy versus placebo, with a favorable safety and tolerability profile, supporting its further investigation in late‐stage trials.

**Disclosure:** Funded by SIMR. B. Fan reports no conflicts of interest. All other authors are employees of SIMR, including S. Li, the CEO, who participated in study oversight and approval. The funder was involved in study design, data analysis and manuscript preparation.

## LB_17

### Robotic‐assisted rehabilitation accelerates neuropsychological recovery in Ukrainian military personnel with war‐related upper limb injuries

#### I. Halabitska ^1^, I. Kamyshna^1^, P. Petakh^2^, O. Kamyshnyi^1^

##### ^1^I. Horbachevsky Ternopil National Medical University, Ukraine, ^2^Uzhhorod National University, Ukraine

**Background and aims:** Upper limb injuries and amputations in military personnel are associated with PTSD, anxiety, depression, and cognitive impairment. While standard rehabilitation is effective, the additional effects of robotic systems require further study.

**Methods:** Sixty military personnel with upper limb amputations were enrolled. Group 1 (*n* = 30) received standard rehabilitation, while Group 2 (*n* = 30) additionally underwent robotic therapy (DIEGO®). Assessments at baseline, 14, 30, and 120 days included PCL‐5, GAD‐7, PHQ‐9, and MoCA. Data were analyzed using repeated‐measures ANOVA, Cohen's d, and η^2^.

**Results:** At baseline, both groups had high psychoemotional distress and moderate cognitive impairment without significant differences: PCL‐5 58.2 ± 6.1 vs. 57.9 ± 5.8 (*p* = 0.84), GAD‐7 15.3 ± 3.4 vs. 15.1 ± 3.2 (*p* = 0.79), PHQ‐9 17.8 ± 4.0 vs. 18.0 ± 3.8 (*p* = 0.88), MoCA 23.1 ± 2.3 vs. 23.3 ± 2.5 (*p* = 0.67).

PCL‐5 scores improved: day 14 51.6 ± 5.7 vs. 45.9 ± 5.4 (*p* = 0.04), day 30 43.8 ± 5.4 vs. 35.2 ± 4.9 (*p* = 0.003), day 120 34.5 ± 5.2 vs. 26.1 ± 4.8 (*p* = 0.002; d = 1.68; η^2^ = 0.31).

GAD‐7 differences were significant only at day 120: 9.2 ± 2.6 vs. 6.3 ± 2.1 (*p* = 0.004; d = 1.22; η^2^ = 0.24).

PHQ‐9 improvement: day 14 15.0 ± 3.6 vs. 13.6 ± 3.3 (*p* = 0.06), day 30 12.8 ± 3.3 vs. 9.6 ± 2.9 (*p* = 0.01), day 120 10.4 ± 3.1 vs. 7.2 ± 2.5 (*p* = 0.006; *d* = 1.13; η^2^ = 0.21).

MoCA: day 14 23.8 ± 2.2 vs. 25.1 ± 2.0 (*p* = 0.02), day 30 24.4 ± 2.1 vs. 26.3 ± 1.9 (*p* = 0.01), day 120 25.0 ± 2.1 vs. 27.4 ± 1.8 (*p* = 0.001; *d* = 1.25; η^2^ = 0.28).

**Conclusion:** Robotic therapy (DIEGO®) added to standard rehabilitation leads to faster and greater improvement in psychoemotional and cognitive functions, supporting its integration into military rehabilitation.

Supported by the National Research Foundation of Ukraine (Project No. 2025.07/0021).

**Disclosure:** Nothing to disclose.

## LB_18

### GRAND CANYON pivotal cohort of Sevasemten in adults with Becker muscular dystrophy: Rationale, study design and baseline characteristics

#### C. McDonald^1^, R. Roxburgh^2^, E. Mercuri^3^, K. Claeys^4^, M. Guglieri^5^, E. Niks^6^, K. Mathews^7^, A. Connolly^8^, L. Bello^9^, B. Byrne^10^, A. Veerapandiyan^11^, J. MacDougall^12^, R. Dreghici^12^, J. Donovan ^12^

##### ^1^University of California‐Davis, Davis, California, USA, ^2^University of Auckland, Auckland, New Zealand, ^3^Catholic University, Rome, Italy, ^4^Department of Neurology, University Hospitals Leuven, and Laboratory for Muscle Diseases and Neuropathies, KU Leuven, Leuven, Belgium, ^5^John Walton Muscular Dystrophy Research Centre, Newcastle University and Newcastle Hospitals NHS Foundation Trust, Newcastle upon Tyne, England, UK, ^6^Leiden University Medical Center, Leiden, The Netherlands, ^7^University of Iowa, Iowa City, Iowa, USA, ^8^Nationwide Children's Hospital, Columbus, Ohio, USA, ^9^University of Padova, Padova, Italy, ^10^University of Florida, Gainesville, Florida, USA, ^11^Arkansas Children's Hospital, University of Arkansas for Medical Sciences, Little Rock, Arkansas, USA, ^12^Edgewise Therapeutics, Boulder, Colorado, USA

**Background and aims:** There are no currently approved BMD therapies. Sevasemten, an investigational skeletal myosin inhibitor, is designed to protect muscle against contraction‐induced damage in Becker muscular dystrophy (BMD). GRAND CANYON (NCT05291091), a double‐blind, placebo‐controlled cohort, evaluates sevasemten efficacy and safety.

**Methods:** Ambulatory BMD adults were randomized and stratified by NSAA and rise from floor (RFF). Primary endpoint is NSAA change from baseline at 18 months; secondary endpoints are TFTs, stride velocity 95th percentile (SV95C), and MRI fat fraction.

**Results:** 175 participants enrolled (mean age (SD): 33 (9) years; baseline NSAA: 19.6 (7.6); 58% with NSAA < 22). 4‐Stair Climb (4SC), 10‐meter walk run (10MWR), and 100‐m walk run (100MWR) tests were 6.3 (6.1), 8.2 (6.5), and 83.1 (33.8) seconds, respectively. RFF was 5.4 (3.0) seconds. SV95C was 1.2 (0.24) meters/second. Upper limb function was largely maintained, with 15% having entry level PUL 2.0 < 6. In the initial CGI‐S, 51% of participants had moderately or markedly severe disease and 48% reported moderate or severe disease per PGI‐S. Pre‐treatment NSAA strongly correlated with 4SC (*r* = 0.90), 10MWR (*r* = 0.89), 100MWR (*r* = 0.82), RFF (r = 0.87), and SV95C (*r* = 0.83), all *p* ≤ 0.001.

**Conclusion:** GRAND CANYON is the largest BMD interventional trial; results could support approval as the first targeted BMD therapy. Pre‐treatment NSAA correlation analysis provides initial insight into potential alternatives to assessing functional status.

**Disclosure:** The GRAND CANYON trial is sponsored by Edgewise Therapeutics. Craig McDonald, Eugenio Mercuri, Kristl Claeys, Michela Guglieri, Erik H. Niks, Katherine Mathews, Anne M. Connolly, Luca Bello, Barry Byrne, Aravindhan Veerapandiyan are consultants for Edgewise. Jim MacDougall, Roxana Donisa Dreghici, and Joanne Donovan are employees and shareholders of Edgewise Therapeutics. All non‐Edgewise authors are investigators in the GRAND CANYON trial.

